# Role of Intra-Parotid Lymph Node Metastasis in Primary Parotid Carcinoma

**DOI:** 10.3390/life12122053

**Published:** 2022-12-07

**Authors:** Tetsuya Terada, Ryo Kawata

**Affiliations:** Department of Otorhinolaryngology, Osaka Medical and Pharmaceutical University, Takatsuki 569-8686, Osaka, Japan

**Keywords:** parotid carcinoma, intra-parotid lymph nodes, cervical lymph nodes, metastasis

## Abstract

The parotid gland contains intra-glandular lymph nodes, the distribution of which is crucial for understanding the pathogenesis of intra-parotid lymph node metastases of parotid carcinoma and other head and neck carcinomas. Positive intra-parotid lymph node metastasis predicts the risk of positive cervical nodal metastasis. It is important to establish whether prophylactic neck dissection, including intra-parotid lymph nodes, contributes to treatment outcomes. The presence or absence of intra-parotid lymph nodes or metastasis-positive lymph nodes warrants further study. A preoperative diagnosis by imaging and fine-needle aspiration cytology of intra-parotid lymph nodes is difficult. Although intraoperative frozen section biopsy is performed during surgery, it is challenging to identify intra-parotid lymph nodes. The number of lymph nodes was the largest (47%) in the lower half of the superficial lobe, with 35% of nodes being concentrated in the inferior part of the cervicofacial branch, i.e., the lower pole of the parotid gland. Therefore, superficial parotidectomy and lower pole lobectomy need to be performed in cases in which a malignant tumor localizes to the superficial lobe or a lower pole. When intra-parotid lymph node metastases are detected during surgery, selective neck dissection (at least levels II and III) needs to be simultaneously performed.

## 1. Introduction

During the embryonic period, the construction of the lymph node system in the neck is assumed to occur following the encapsulation of the submandibular and sublingual glands, but prior to that of the parotid gland. Therefore, lymph nodes are present throughout the parotid gland, whereas intra-glandular lymph nodes are absent in the submandibular and sublingual glands [1,2].

Similar to periparotid lymph nodes, the parotid gland also contains intra-glandular lymph nodes, the distribution of which is important for understanding the pathogenesis of intra-parotid lymph node metastases of parotid carcinoma and other head and neck carcinomas.

Recent studies on parotid carcinoma [3,4,5,6] examined the rate of metastasis to intra-parotid lymph nodes and positive intra-parotid lymph nodes were identified as an independent risk factor for a poor prognosis in patients with primary parotid cancer. However, a number of issues have yet to be clarified in the treatment guidelines for patients with positive intra-parotid lymph node metastases, such as staging categories and treatment selection. Furthermore, treatment guidelines are needed for primary parotid cancer with positive intra-parotid lymph node metastases.

## 2. Distribution of Lymph Nodes within the Parotid Gland

Although the first study on intra-parotid lymph nodes was by Neisse in 1898 [7], limited information is currently available on not only the number but also the distribution of lymph nodes. Marks [8] examined 17 parotid glands in 1984 and found an average of 3.94 and 1.05 lymph nodes in the superficial parotid lobe and deep lobe, respectively, that 79% of all lymph nodes in the parotid gland were present in the superficial lobe, and also that the distribution of lymph nodes within each lobe showed no localization bias. McKean et al. [9] examined 20 parotid glands in 1985 and found 8.85 lymph nodes in the superficial lobe and 0.8 in the deep lobe, with 92% of nodes being present in the superficial lobe. Garatea-Crelgo et al. [10] analyzed 60 parotid glands in 1993 and found 3.2 nodes in the superficial lobe and 1.2 nodes in the deep lobe, with 73% of nodes being present in the superficial lobe. Ergün et al. [11] examined 84 parotid glands in 2014 and found intra-glandular lymph nodes in the superficial lobe in 95% of cases and in the deep lobe in 31% of cases; 2.97 nodes were present in the superficial lobe and 0.41 nodes in the deep lobe, and lymph nodes were within the superficial lobe in 88% of all cases.

In a recent study by Sonmez et al. [12] that used the course of the facial nerve as the boundary, 128 parotid glands were divided into the superficial and deep lobes, and an assumed line extending from the bifurcation of the upper and lower main branches of the facial nerve to Stenon’s duct was established. The superficial and deep lobes have been divided into upper and lower halves, respectively, with this line as the boundary [13,14].

The distribution of lymph nodes in seven regions, including the accessory lobe, has been investigated, and the findings obtained showed an average of 6.11 lymph nodes in the entire superficial lobe and 0.98 in the deep lobe, with 86% of nodes in the superficial lobe. Furthermore, the number of lymph nodes (47%) was the highest in the lower half of the superficial lobe, with 35% of nodes being concentrated in the inferior part of the cervicofacial branch, i.e., the lower pole of the parotid gland. Intra-glandular lymph nodes of the deep lobe accounted for only 14% of the total, of which 9% were in the lower half and 5% in the upper half, indicating that intra-parotid lymph nodes were more common within the superficial lobe, accounting for approximately 70–90% of the total. They were also considered to be more common in the lower part of the superficial lobe, particularly in the lower pole. The number of intra-glandular lymph nodes was also independent of age, sex, and BMI.

## 3. Implications of Intra-Glandular Lymph Node Metastasis

Regarding the indications of prophylactic lymphatic dissection for parotid cancer, it is important to consider the presence of lymph nodes within the parotid gland, mainly in the superficial lobe and lower pole, and lymphatic flow through intra-parotid lymph nodes towards cervical lymph nodes. The extent of neck dissection and parotidectomy for parotid cancer needs to be discussed in more detail in the future and depends on the presence or absence of neck and intra-parotid lymph node metastases. Positive intra-parotid lymph node metastasis has been identified as a predictor of an increased risk of positive cervical nodal metastasis.

The rate of intra-parotid lymph node metastases in parotid carcinoma is between 7.6 and 73.3%, with a wide range of positive metastatic rates being reported [15]. This wide variation indicates the need for further studies on intra-parotid lymph node metastasis rates. Chwee et al. [5] showed that cases with negative clinical node metastasis, but positive intra-parotid node metastasis had worse outcomes and a higher risk of locoregional recurrence. Yanping et al. reported that the positive rate of intra-parotid lymph node metastasis in parotid carcinoma was 32.9%, which correlated with the T stage, and also that the prognosis of patients deteriorated when the number of intra-parotid metastatic lymph nodes was two or more [6]. However, the presence or absence of intra-parotid lymph node metastases is currently not considered in the UICC staging system for staging categories or treatment selection.

Previous studies demonstrated that cases with positive intra-parotid lymph nodes had a lower five-year survival rate [16,17,18,19,20], and the presence or absence of intra-parotid lymph node metastases was an important prognostic factor. Mucoepidermoid cancer is one of the most common malignant tumors in high-grade parotid cancers [21,22]. Additionally, the 5-year recurrence-free survival rate is 65%, and the prognosis is considered representative of all high-grade parotid carcinomas. Although tumor size, cervical lymph node metastasis, tumor grade, and nerve invasion are commonly discussed prognostic factors for parotid carcinoma [23], the relevance of intraparotid lymph node metastasis has rarely been evaluated.

Xue et al. studied highly malignant cases of mucoepidermoid carcinoma of the parotid gland and stated that intraparotid lymph node metastasis, especially in the deep lobes of the parotid gland, is associated with a lower recurrence-free survival rate [24].

Additionally, they discussed that the reason for this is that the deep lobe of the parotid gland is adjacent to the parapharyngeal space and is rich in lymphatic vessels, making it easy to recur after cervical neck dissection, and the lymph nodes in the deep lobe may have remained after superficial parotid lobectomy, resulting in recurrence.

However, a preoperative diagnosis by imaging and fine-needle aspiration cytology of intra-parotid lymph nodes is difficult. Although intraoperative frozen section biopsy is performed during surgery, it is often challenging to identify intra-parotid lymph nodes. Therefore, superficial parotidectomy and lower pole resection (lower pole lobectomy) are recommended for cases in which a malignant tumor localizes to the superficial lobe or a lower pole. When intra-parotid lymph node metastases are detected during surgery, selective neck dissection (at least levels II and III) needs to be simultaneously performed.

Furthermore, it is important to establish whether prophylactic neck dissection, including intra-parotid lymph nodes, contributes to treatment outcomes. Therefore, the presence or absence of intra-parotid lymph nodes or metastasis-positive lymph nodes needs to be investigated in detail.

## 4. Assessment of Intra-Parotid Lymph Nodes in the UICC Staging System

The prognostic potential of the patterns of lymph node metastasis in parotid malignancies remains unclear. Cervical nodes and intra-parotid lymph nodes are not clearly differentiated in the current UICC staging system for local lymph node metastasis (N stage). For example, can an intra-parotid metastatic lymph node less than 3 cm but without metastasis to the lateral neck be classified as N1 [25]?

Intra-parotid lymph node metastasis has been identified as a poor prognostic factor in non-malignant melanoma skin cancer and is of prognostic significance [26]. Although positive periparotid lymph nodes are associated with a poor prognosis in patients with primary malignant tumors of the parotid gland [27], few studies have assessed the prognostic impact of intra-parotid lymph node metastasis. However, recent studies revealed a relationship between positive intra-parotid lymph node metastasis in parotid malignancies and a poorer clinical outcome [4,5].

There are currently no established treatment strategies, including staging, for patients with positive intra-parotid lymph nodes. For example, a patient with intra-parotid lymph node metastases, but not cervical lymph node metastases, classified as N0 according to the current staging system may need to be assessed as N1 and have an appropriate treatment plan considered. Treatment guidelines need to be developed for primary parotid cancer with intra-parotid lymph node metastases.

## 5. Locations of Parotid Malignancies and Intra-Parotid Lymph Node Metastasis

Many patients with malignant tumors of the parotid gland do not present with intra-parotid lymph node metastases. Therefore, the first lymph node of regional metastasis may not be an intra-parotid lymph node in all cases of parotid cancer. Since the lymphatic flow in the parotid gland runs from above to below, the location of the primary tumor may influence the presence or absence of intra-parotid lymph node metastasis.

In consideration of the location of parotid tumors, tumors are often located in the inferior lobe. Parotid tumors in the inferior lobe often present with level IIa or IIb lymph node metastases rather than intra-parotid lymph node metastases, possibly because lymphatic flow within the parotid gland is directed from above to below. The metastatic route of a parotid malignancy is assumed to involve the flow of lymph within the parotid gland and the location of the parotid tumor; however, the underlying mechanisms currently remain unclear and warrant further study.

## 6. Intra-Parotid Node Metastases: An Indicator of Prognosis

Since the optimal treatment for N0 neck cancer in parotid carcinoma remains controversial, the presence or absence of intra-parotid lymph node metastasis has potential as a marker in decision-making for elective neck dissection. However, preoperative evaluations of intra-parotid nodes based on images, such as ultrasound, CT, and MRI, have more limitations than those of cervical lymph node metastasis. Although an intraoperative diagnosis using frozen sections is often performed for cervical lymph nodes to select the indication of neck dissection, difficulties are associated with the detection and extraction of intra-parotid nodes in the gland parenchyma. Theoretically, when positive nodes are detected in the cervical/intra-parotid area, neck dissection is indicated for primary parotid cancer. 

Wu et al. [18] retrospectively analyzed the predictive value of intra-parotid nodal metastases in patients undergoing neck dissection for parotid mucoepidermoid cancer. The overall intra-parotid metastasis rate was 25% (30/122) and seven patients had lymph node metastasis in the deep lobe of the parotid gland, which correlated with the stage, cervical lymph node metastasis, perineural invasion, lymphovascular invasion, and the pathological grade. They also reported that positive nodes in the intra-parotid region were associated with an increased risk of the recurrence of parotid cancer. A relationship has been reported between intra-parotid nodal metastases and clinicopathological variables [4,5,28], and widely accepted risk factors were also identified in the present study. These findings provide support for the hypothesis that intra-parotid nodes need to be interpreted as sentinel nodes [5]. In other words, intra-parotid nodes are potentially predictive of occult neck lymph node metastases.

The predictive value of intra-parotid lymph node metastasis has also been investigated for cutaneous squamous cell cancer. O’Brien et al. [22] identified metastasis to intra-parotid lymph nodes in patients with cutaneous squamous cell cancer as an independent predictor of a poorer outcome. Although factors to predict the outcome of mucoepidermoid cancer have been widely examined in patients with parotid cancer [29,30], the predictive value of intra-parotid metastases remains unclear. To the best of our knowledge, Lim et al. [5] were the first to report that local recurrence was more likely in patients with clinical N0 neck and intra-parotid lymph node metastases than in those without intra-parotid lymph node metastases. They reported that the patients with positive intraparotid lymph nodemetastasis had a higher 3-year disease-specific mortality compared to those without intraparotid lymph node metastasis (75% VS 34.2%; *p* = 0.0037).

Klussmann et al. [4] identified intra-parotid lymph node metastasis as an additional risk factor for tumor recurrence in 55 patients with intra-parotid lymph node metastasis, while Nisa et al. [20] showed that the disease-free survival rate was reduced in patients with intra-parotid node metastasis. 

## 7. Importance of Collaborations with Pathologists

Future research needs to focus on the histopathological criteria that define evaluations of intra-parotid lymph nodes in histopathology reports. The most important issue is that not only surgeons but also pathologists are not familiar with intra-parotid lymph nodes. Furthermore, it is technically challenging to detect these nodes grossly even after resection of the parotid gland. Surgeons who treat parotid tumors need to request that pathologists perform a routine search on all parotid specimens for intra-parotid lymph nodes. Consensus reports on the histopathology of parotid tumors generally indicate the importance of cervical lymph node metastases; however, the current TNM staging system does not include any guidelines on the treatment of metastasis-positive lymph nodes within the parotid gland [31]. (Sub)total parotidectomy at the very least is recommended for high-grade parotid carcinoma by the majority of guidelines [32,33], and deep lobe removal is the most effective procedure for removing potential metastasis to the deep parotid nodes [33].

## 8. Importance of Evaluations of Intra-Parotid Lymph Nodes in the Local Control of Parotid Carcinoma

Yanping et al. [6] showed that the intra-parotid lymph node metastasis rate was high in patients with parotid carcinoma and correlated with grades and T stages. They also found a poor local control rate in patients with more than two intra-parotid metastatic lymph nodes. The N factor, which indicates the classification of lymph node metastasis, refers to nodes for regional and cervical sites, and intra-parotid lymph node metastases are not included in the N classification, which may be an important issue in future studies to predict the local control rate of parotid cancer.

## 9. Intra-Parotid and Cervical Lymph Node Metastasis in Primary Parotid Carcinoma

Elective neck dissection in the cervical region is indicated for parotid carcinoma with N0 advanced T stage/high grade cancer, and the simultaneous resection of intra-parotid lymph nodes with (sub)total parotidectomy is recommended. However, limited information is currently available on lymph node metastasis in early stage parotid carcinoma. Stenner et al. [34] examined the occurrence of lymph node metastases and its impact on local control and survival rates in patients with T1 and T2 carcinomas and proposed total parotidectomy as the most appropriate procedure to remove intra-parotid lymph nodes showing a high rate of occult neck metastases, even in T1/T2 cases. Lymph node metastasis to the neck is one of the most important factors in therapy for and the prognosis of parotid carcinoma [35,36,37]. Parotid carcinoma metastasizes not only to the lymphatic surroundings of the parotid gland and cervical region but also to intra-parotid lymph nodes [34]. Intra-parotid node metastases were previously detected in 73.3% of pN+ patients. [34]. Kouka et al. [38] described that a large population-based study on patients with primary parotid carcinoma confirmed that intraparotid lymph node metastasis is an indicator of worse overall survival. They recommended that a standardized assessment of intraparotid lymph nodes after parotid cancer surgery is always performed and including the assessment of the intraparotid lymph nodes in the N classification of UICC tumor staging should be considered.

## 10. Surgery for Parotid Carcinoma from the Viewpoint of Intra-Glandular Lymph Nodes—Lower Half Resection 

### 10.1. Concept

Partial lobectomy is the basic procedure for benign tumors, but it is also a good indication for relatively small low- to intermediate-grade malignancies. However, partial lobectomy may be appropriate for local cancer resection, but it does not consider intra-glandular lymph node metastasis [15]. From this perspective, lobectomy may be the appropriate extent of resection for even small tumors [39]. Although a total parotidectomy for small tumors would solve this problem [40], a total parotidectomy that preserves all facial nerves is not an easy procedure. We have proposed a lower hemi resection of the parotid gland procedure considering the distribution of lymph nodes within the parotid gland and lymphatic flow. In terms of tumor location, 1/3 to 1/4 of parotid carcinomas are located in the lower pole [41]. In 1102 cases of benign parotid tumors operated on in our department, 23.1% were tumors of the inferior pole [42]. The majority of intra-parotid lymph nodes are located in the superficial lobe and the inferior pole, so the idea is to resect the same area. If the tumor is located in the inferior pole, a lower lobectomy alone is acceptable, but if it is located in the superficial lobe (upper pole), a superficial lobectomy is also performed.

### 10.2. Indications

When the indication for lower lobectomy is based on the grade of parotid carcinoma, low- to intermediate-grade carcinoma is a good indication. As a rule, the facial nerve must not be invaded. The best indication is when the tumor is localized to the lower pole, in which case a lower hemi resection is performed. If the carcinoma is confined to the superficial lobe of the upper pole, a lobectomy is performed in addition to a lower lobectomy. Considering the relationship to the facial nerve, the size of the tumor should be limited to T1 and part of T2. In the case of high-grade carcinoma, the indications for lower lobectomy are even more limited, since high-grade carcinoma often involves severing the facial nerve. Tumors must be confined to the inferior pole or superficial lobes which do not invade the facial nerve.

### 10.3. Surgical Technique

**Figure 1:**
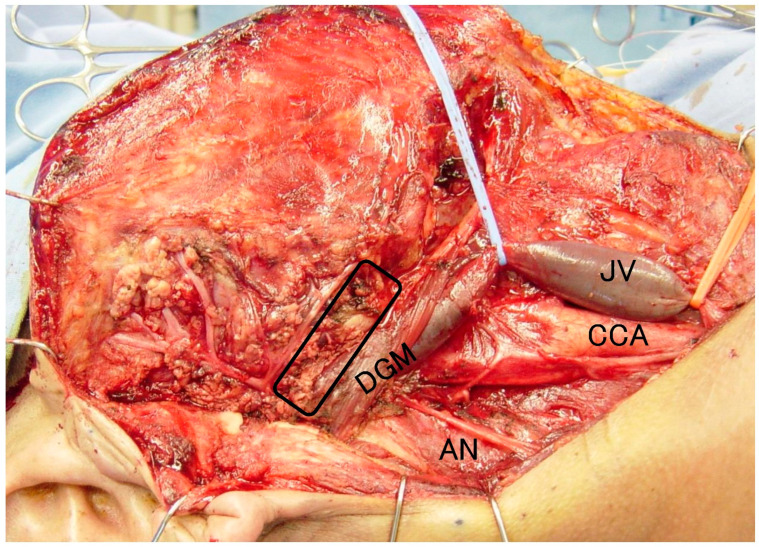
A superficial lobectomy and cervical neck dissection for parotid carcinoma. The black frame shows the area of the lower half of the resection caudal to the mandibular border branch of the facial nerve.

In the lower half of the resection, the main trunk of the facial nerve is first identified and then the inferior main branch and the mandibular border branch are dissected. After dissecting the entire circumference of the inferior main branch and mandibular border branch, they can be moved approximately 1 cm upward (cephalad side). The parotid gland is removed inferiorly (caudally) while protecting the same area (Figure 1). Figure 1 shows a superficial lobectomy and cervical neck dissection for parotid carcinoma. The black frame shows the area of the lower half of the resection caudal to the mandibular border branch of the facial nerve. The parotid gland should be resected vertically toward the depth. We reviewed 184 cases of parotid adenocarcinoma operated on in our department and 10 cases of lower lobectomy (2 high malignant, 8 low/intermediate malignant).

## 11. Conclusions

The parotid gland consists of between 3 and 10 lymph nodes, approximately 70% of which are present in the superficial lobe. Although the current TNM staging system does not include intra-parotid lymph node metastases, its potential as a prognostic factor has been suggested.

The N factor in the TNM staging system, which indicates the classification of lymph node metastasis, refers to nodes for regional and cervical sites and does not include intra-parotid lymph node metastases. This may be an important issue in predictions of the local control of parotid cancer. Furthermore, since local recurrence is more likely in patients with clinical N0 neck and intra-parotid lymph node metastases than in those without intra-parotid lymph node metastases, surgeons need to request that pathologists perform a routine search on parotid samples for intra-parotid nodal metastases.

When intra-parotid lymph node metastases are detected during surgery, selective neck dissection (at least levels II and III) needs to be simultaneously performed. Since the optimal treatment for N0 neck cancer in parotid carcinoma remains controversial, the presence or absence of intra-parotid lymph node metastasis may be used as a marker in decision-making for elective neck dissection (Table 1).

## Figures and Tables

**Table 1:** Conclusion statement.

Intra-Parotid Lymph Nodes
Known facts
There are 3–10 lymph nodes within the parotid gland Approximately 70% are located in the superficial lobe Intra-parotid lymph node metastases is not considered in the TNM classification Has potential as a prognostic factor
Need to know and consider
The significance of intra-parotid lymph node metastasis as an N factor in TNM Collaboration with pathologists for intra-parotid lymph nodes Effects on recurrence and treatment outcomes The adjunctive role of cervical dissection and selecting the extent of dissection

## Abbreviations

JV: Juglar vein, CCA: Common carotid artery, AN: Accessory nerve, DGM: Digastric muscle.
